# Magnesium Oxide Nanoparticles Reinforced Electrospun Alginate-Based Nanofibrous Scaffolds with Improved Physical Properties

**DOI:** 10.1155/2017/1391298

**Published:** 2017-06-11

**Authors:** R. T. De Silva, M. M. M. G. P. G. Mantilaka, K. L. Goh, S. P. Ratnayake, G. A. J. Amaratunga, K. M. Nalin de Silva

**Affiliations:** ^1^Nanotechnology and Science Park, Sri Lanka Institute of Nanotechnology (SLINTEC), Pitipana, Homagama, Sri Lanka; ^2^School of Mechanical and Systems Engineering, Newcastle University, Newcastle Upon Tyne, UK; ^3^Electrical Engineering Division, Department of Engineering, University of Cambridge, 9 J. J. Thomson Avenue, Cambridge CB3 0FA, UK; ^4^Department of Chemistry, University of Colombo, Colombo 3, Sri Lanka

## Abstract

Mechanically robust alginate-based nanofibrous scaffolds were successfully fabricated by electrospinning method to mimic the natural extracellular matrix structure which benefits development and regeneration of tissues. Alginate-based nanofibres were electrospun from an alginate/poly(vinyl alcohol) (PVA) polyelectrolyte complex. SEM images revealed the spinnability of the complex composite nanofibrous scaffolds, showing randomly oriented, ultrafine, and virtually defects-free alginate-based/MgO nanofibrous scaffolds. Here, it is shown that an alginate/PVA complex scaffold, blended with near-spherical MgO nanoparticles (⌀ 45 nm) at a predetermined concentration (10% (w/w)), is electrospinnable to produce a complex composite nanofibrous scaffold with enhanced mechanical stability. For the comparison purpose, chemically cross-linked electrospun alginate-based scaffolds were also fabricated. Tensile test to rupture revealed the significant differences in the tensile strength and elastic modulus among the alginate scaffolds, alginate/MgO scaffolds, and cross-linked alginate scaffolds (*P* < 0.05). In contrast to cross-linked alginate scaffolds, alginate/MgO scaffolds yielded the highest tensile strength and elastic modulus while preserving the interfibre porosity of the scaffolds. According to the thermogravimetric analysis, MgO reinforced alginate nanofibrous scaffolds exhibited improved thermal stability. These novel alginate-based/MgO scaffolds are economical and versatile and may be further optimised for use as extracellular matrix substitutes for repair and regeneration of tissues.

## 1. Introduction

Polymeric nanofibres have gained enormous attention in the recent past due to those of particular interest in tissue engineering applications [1]. Typically, the artificial scaffolds which are being used in tissue engineering applications should mimic the spatial-porous-structured morphology of extracellular matrices (ECM) which can be found in native tissues and organs of human body to facilitate the cell growth and proliferation. Artificial ECM scaffolds which are used for tissue engineering applications can be fabricated using different techniques such as freeze-drying [2, 3], template-based solution casting [4], 3D printing [5], wet-spinning [6], and electrospinning [1, 3]. Among these techniques, electrospinning is one of the most feasible methods to produce scaffolds due to its versatility and robustness.

Electrospinning enables the production of three-dimensional porous-structured fibrous mat which mimics the natural structure of ECM and helps to promote cell adhesion and permit sufficient gases to exchange [1]. Recently, biopolymer based electrospun scaffolds have been extensively studied for tissue engineering applications. To date, a number of biopolymers, notably chitosan [7], alginate [8], poly(lactic acid) (PLA) [9], and poly(ethylene oxide) (PEO) [10], have been used in fabricating nanofibrous scaffolds by electrospinning. In particular, alginate takes a predominant place due to its biocompatibility, biodegradability, and relatively low cost for mass production [11]. These unique properties have enabled alginate to be used in many biomedical applications such as drug delivery and skin/bone scaffolds [11, 12]. Alginate is a linear polysaccharide copolymer composed of two sterically different repeating units of *β*-d-mannuronate (M unit) and *α* -L-glucuronate (G unit) in various M/G ratios. Although a few aspects of alginate-based electrospun scaffolds related to ECM tissues such as cell adhesion [13], alterations in scaffold fibre dimensions [14] and scaffold degradation [15] have been extensively studied, and only a limited number of studies have been carried out on reinforced alginate nanofibrous mats to ensure the required mechanical strength and structural properties.

Typically the mechanical properties of biopolymer scaffolds are enhanced by either cross-linking or incorporating micro-/nanofillers. Although cross-linking biopolymer scaffolds is a promising method, it reduces the in vivo degradation rate of the biopolymeric scaffold and changes the host tissue responses [16]. On the other hand, incorporating micro-/nanofillers into polymeric fibres enables the production of multifunctional scaffolds with enhanced mechanical properties and other vital characteristics such as antimicrobial and anti-inflammatory characteristics of ECM scaffolds. To date, different types of micro-/nanofillers such as hydroxyapatite (HA) [12, 17], chitin whiskers [18], ZnO [19], and Ag nanoparticles [20] have been used to reinforce electrospun alginate nanofibrous scaffolds. With the aforementioned requirements in reinforcing alginate nanofibrous scaffolds, it is essential to widen the research scope by evaluating different types of nanofillers to enhance the mechanical strength of alginate scaffolds. In this study MgO nanoparticles have been utilized for the first time to reinforce electrospun alginate fibrous scaffolds and their performances were evaluated.

MgO nanoparticles have gained much interest in recent years due to their attractive properties including large surface area-to-volume ratio, thermal and electrical insulation, strong adsorption ability of dye wastes and toxic gases, antimicrobial activity, nontoxicity, and biocompatibility [21–25]. With these outstanding features, MgO nanoparticles have been vastly used in applications such as a catalyst, ceramic material, thermal and electrical insulator, bactericide, material to treat toxic liquid and gaseous wastes, multifunctional composites, and a refractory material [21–25]. MgO nanoparticles are conveniently synthesised with economical routes using low-cost raw materials including magnesium salts, brines, and naturally occurring minerals such as dolomite and magnesite [23]. MgO nanoparticles are mainly synthesised by calcination of nanometre scale magnesium carbonates, magnesium hydroxide, and their composites. In the calcination method, precursor nanoparticles are basically kept in the nanometre scale using polymers and surfactants [22, 23, 25, 26]. Recently MgO nanoparticles have been used to reinforce a number of biopolymers. Zhao et al. fabricated MgO nanowhiskers reinforced PLA nanocomposites films for bone repair and fixation [27]. In another study, chitosan was reinforced with spherical MgO nanoparticles for high performance packaging applications [21]. For instance, tensile stress and elastic modulus significantly improved by 86% and 38%, respectively, with the addition of 5% (w/w) of MgO into chitosan matrix.

In this study, MgO nanoparticles reinforced alginate nanofibrous scaffolds were fabricated by electrospinning method for the first time and their mechanical and structural properties were systematically investigated. Spherical MgO nanoparticles were synthesised using a polymer template-based ex situ method. Herein, the influence of MgO nanoparticles on the mechanical, morphological, chemical, and thermal properties of alginate nanofibrous scaffolds was investigated. Furthermore, tensile and structural properties of MgO reinforced alginate scaffolds were compared with those of the glutaraldehyde cross-linked alginate scaffolds. The fabricated MgO reinforced alginate nanofibrous scaffolds exhibited a great potential to be used as an artificial scaffold to substitute extracellular matrices.

## 2. Materials and Methods

### 2.1. Materials

Sodium alginate powder and poly(vinyl alcohol) (PVA) (with a *M*_*w*_ of 89,000), acrylic acid (AA) (99% purity), potassium persulfate (99% purity), magnesium chloride hexahydrate (99% purity), and sodium hydroxide (99% purity) were used in this study and purchased from SRL Ltd.

### 2.2. Synthesis of MgO Nanoparticles

MgO nanoparticles used in this study were synthesised using a method reported by Mantilaka et al. [23] with some modifications. In the current method, poly(acrylic acid) (PAA) was prepared by polymerization of 25 mL of 0.5 M AA using 1 g of K_2_S_2_O_8_ initiator in an aqueous medium. PAA was added to 100 mL of 1 M NaOH solution. 25 mL of 0.5 M MgCl_2_ was added dropwise to the PAA/NaOH mixture while stirring to produce PA^−^ stabilized Mg(OH)_2_ precursor nanoparticles. Finally, the precursor was heat-treated at 600°C for 3 h to produce MgO nanoparticles.

### 2.3. Fabrication of Alginate/MgO Nanofibrous Scaffolds by Electrospinning

Electrospun alginate fibrous mats were prepared using an alginate solution which comprises a secondary polymer, PVA. 2% (w/v) alginate solution was prepared by dissolving alginate in distilled water and corresponding amount of MgO was incorporated under vigorous stirring to prepare 10% (w/w) MgO composition (MgO amount is with respect to the weight of alginate). PVA solution of 10% (w/v) was prepared by dissolving PVA in distilled water at 80°C with continuous stirring for 3-4 h. These alginate/MgO and PVA solutions were mixed together in 3 : 2 weight ratio for 4 h under vigorous stirring and followed by an ultrasound treatment (at an amplitude of 80 Hz for 30 min) to achieve a homogeneous MgO dispersion. The resultant solution was electrospun in a horizontal electrospinning setup. All samples were electrospun with a solution flow rate of 8–10 *μ*L/min, having needle to collector distance of 10 cm, needle diameter of 0.5 mm, and voltage of 26–28 kV (electrospinning parameters are given in Table 1). Alginate/PVA (3 : 2 weight ratio) nanofibres (henceforth, the electrospun alginate/PVA (3 : 2 weight ratio) nanofibres are referred to as alginate nanofibres) without MgO nanoparticles were also fabricated by electrospinning for the comparison purposes. Section S2 (prediction parameters are given in Table S2 in Supplementary Material available online at https://doi.org/10.1155/2017/1391298) describes a numerical method to determine the required MgO loading to reinforce alginate fibres (predetermined amount of 10% (w/w) was selected based on that (Fig. S3)).

Additionally, to compare the effects of particle reinforcement with chemical cross-linking, electrospun alginate fibrous mats were cross-linked by immersing those in 20 ml of 2% (v/v) glutaraldehyde solution for 2 h and samples were dried in vacuum oven at 40°C for 24 h. Over-cross-linking led to disrupted fibre structure (Fig. S2).

### 2.4. Characterization of MgO Nanoparticles and Alginate/MgO Scaffolds

#### 2.4.1. Morphological Analysis

The morphologies of the electrospun alginate nanofibrous scaffolds as well as the synthesised MgO nanoparticles were examined using a field-emission scanning electron microscope (FE-SEM) (Hitachi SU6600). To prevent electrostatic charging during observation, the samples were coated with a thin layer of gold. The extent of the impregnation of MgO nanoparticles within the nanofibres was determined by carrying out energy-dispersive X-ray (EDX) spectroscopy with a scanning rate of 192000 CPS for 4.5 min. Surface roughness of the fibres was determined by an atomic force microscope (AFM) (Park Systems, XE-100) using the cantilever mode (10 nm tip radius) at 0.5 Hz frequency. Crystallographic structure of synthesised MgO nanoparticles was analysed using X-ray diffractometer (Bruker, Focus D8). The CuK*α* radiation source was operated at a 40 kV power and 40 mA current and data collected within 20–70° of diffraction angle (2*θ*).

#### 2.4.2. Fourier Transform Infrared (FTIR) Analysis

FTIR spectroscopy (Bruker Vertex 80) was conducted to identify the presence of polymer phases, filler-matrix interfacial interaction, and chemical homogeneity of the electrospun alginate nanocomposites. The results were also compared with those derived from raw PVA and MgO. All spectra were obtained within 500–4000 cm^−1^ with 32 scans per measurement at 0.4 cm^−1^ resolution.

#### 2.4.3. Tensile Test

Mechanical properties such as tensile strength (*σ*), elastic modulus (*E*), and elongation at break (*ɛ*) of alginate/MgO nanocomposites were evaluated using an Instron Tensile test rig, following a procedure in accordance with the ASTM D882-02. A strain rate of 5 mm/min was used in this test. The force-displacement data was evaluated to determine the stress-strain data; here stress and strain are defined as the nominal stress and strain. In particular, to determine the nominal cross-sectional area of the specimen, the width and thickness of each scaffold specimen were measured using a digital micrometer screw-gauge (Mitutoyo, 0.001 mm resolution) prior to testing.

One-way analysis of variance (ANOVA) was implemented, complemented by the Tukey Post Hoc test, using commercial software (OriginPro 8) to investigate for significant difference in the respective *σ*, *E*, and *ɛ* among the three different groups, namely, alginate scaffolds, alginate/MgO scaffolds, and cross-linked alginate scaffolds.

#### 2.4.4. Thermal Properties

The thermal decomposition temperature of electrospun alginate nanocomposites was determined by thermogravimetric analysis (TGA) (STD Q600) from 25 to 800°C at a heating rate of 10°C/min in nitrogen medium.

## 3. Results and Discussion

### 3.1. Characteristics of Synthesised MgO Nanoparticles

**Figure 1:**
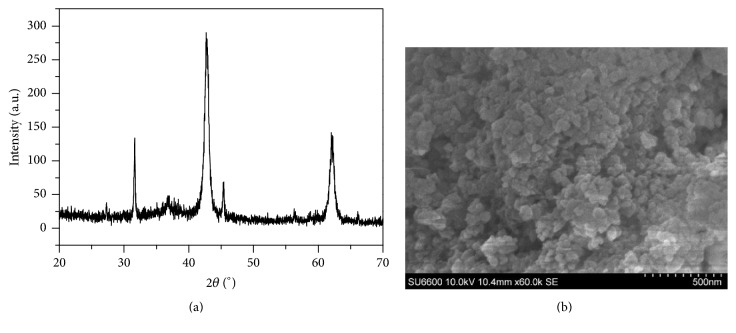
Structural analysis of MgO nanoparticles: (a) a graph of intensity versus angular position derived from XRD analysis and (b) a SEM image of MgO nanoparticles.

Figure 1(a) shows the graph of intensity versus angular position to describe the XRD pattern of synthesised MgO nanoparticles. Of note, the peak positions at 2*θ* = 31.34°, 36.78°, 42.73°, 45.08°, and 62.17° are attributed to the periclase crystalline form of MgO (JCPDS Card Number 75-1525). Any other crystalline phase is not identified in XRD pattern of synthesised MgO nanoparticles. The mean crystallite size of MgO nanoparticles is approximately 23 nm as calculated using the Debye-Scherrer formula. SEM image (Figure 1(b)) of synthesised MgO nanoparticles reveals that the particles are in spherical morphology with an average particle diameter of 45 nm.

### 3.2. Physicochemical Properties of Electrospun Alginate/MgO Nanocomposite Scaffolds

#### 3.2.1. Morphological Properties

**Figure 2:**
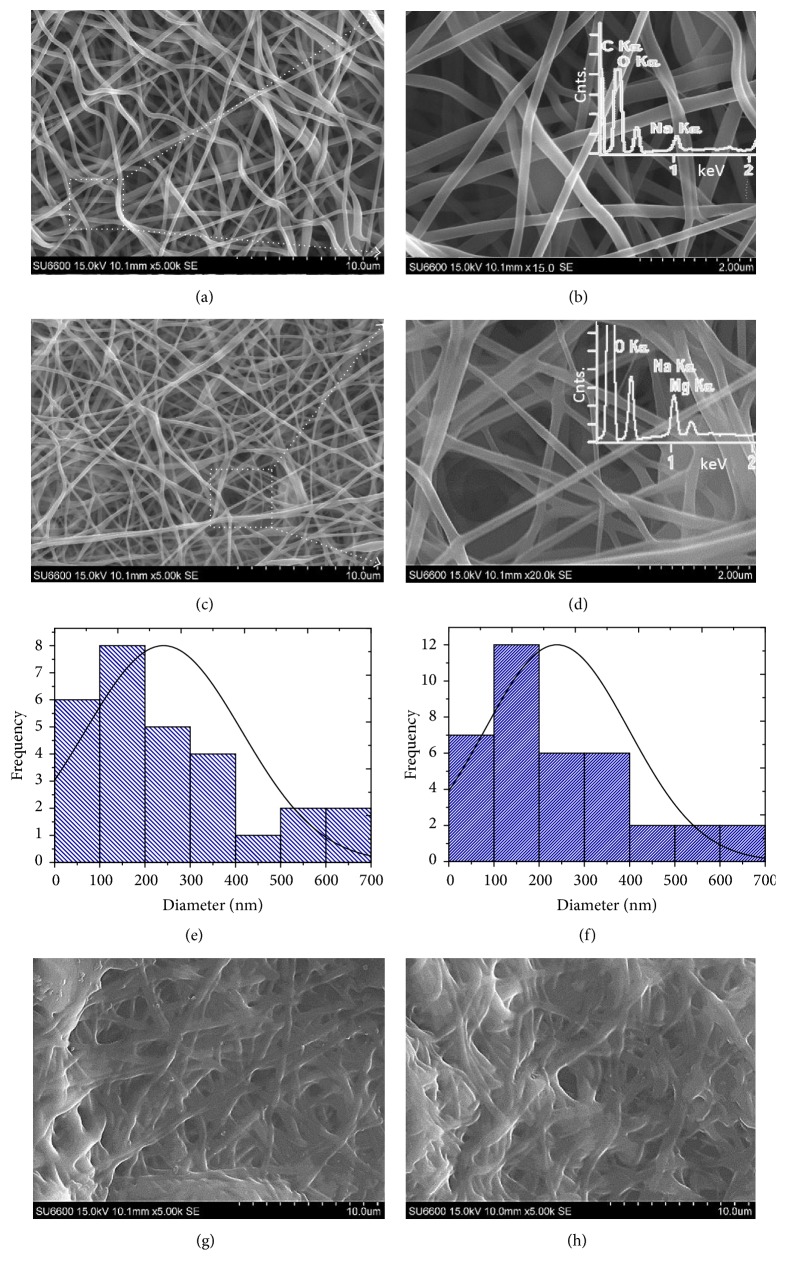
Morphology of electrospun alginate-based scaffolds. (a, b) show SEM images of the alginate-based scaffolds. (c, d) show SEM images of the alginate/MgO scaffolds (with 10% (w/w) MgO). Histograms of frequency versus fibre diameter for the (e) alginate-based fibres and (f) alginate/MgO fibres. (g, h) show SEM images of the cross-linked alginate scaffolds. Insets in (b) and (d) are the graphs of the number of counts versus energy derived from EDX analysis.

Figures 2(a)–2(d) show SEM images of the electrospun alginate nanofibrous scaffolds, revealing uniform, ultrafine, and randomly oriented alginate nanofibres. Of note, the electrospun alginate nanofibrous scaffold appears white (Fig. S1 in supplementary data); all meshes were fabricated to a thickness ranging from 20 to 50 *μ*m. We can estimate the size of the interfibre spacing by examining these SEM images. It is predicted that, to order of magnitude, the pore size ranges from 2 to 50 *μ*m, in good order of magnitude agreement with the results of other types of electrospun scaffold, for example, collagen/PCL/TCP mesh [28]. Insets in Figures 2(b) and 2(d) show the graphs of the number of counts versus energy derived from EDX spectroscopy analysis of the respective alginate scaffolds and alginate/MgO scaffolds. These graphs reveal the presence of Na and Mg peaks corresponding to the alginate and MgO nanoparticles, respectively.

Figures 2(e) and 2(f) show histograms of frequency versus fibre diameters (thickness) derived from a simple image analysis of the electron micrographs of alginate scaffolds and alginate/MgO scaffolds, respectively. This analysis reveals that the alginate-based fibres possess diameters ranging from 62 to 180 nm while alginate/MgO fibres possess diameters of 83–230 nm. Noting that the ranges overlap somewhat, numerically, this suggests that the alginate-based fibres and alginate/MgO fibres do not differ appreciably, valid to order of magnitude (Figure 2(f)). Additionally, it can also be seen that the incorporation of MgO yields no appreciable change in the overall structure of the fibrous scaffolds. The diameter of collagen fibrils ranges from 50 to 350 nm in tendon [29], 20–160 nm in ligament [30], and 30–400 nm in collagen fibril extracts from peristomial membrane of sea urchin (*Paracentrotus lividus*) [31]. Thus, it is seen that the range of values for fibril diameters of the alginate-based/MgO scaffolds overlaps considerably with those in biological tissues. In acellular dermal matrix (ADM) for application as ECM scaffolds, it is found that the diameter of the collagen fibrils ranges from 56.0 ± 8.2 to 60.8 ± 1.9 nm (mean ± SD) from bovine of varying age groups [32]. Thus, it is seen that the lower limit of the fibres diameter of the alginate-based/MgO meshes is in good order of magnitude agreement with those of acellular dermal matrices.

On the other hand, the cross-linked alginate-based nanofibres are densely packed, fused, and appreciably enlarged (Figures 2(g) and 2(h)). Altogether, these contribute to an appreciable reduction in the interfibre porosity of the mesh. To order of magnitude, the pore size is estimated at around 10 *μ*m or lower. Cross-linking with glutaraldehyde results in acetal bridges, which refer to intramolecular and intermolecular interactions of the hydroxyl groups of PVA with the carbonyl groups of glutaraldehyde [33, 34]. Interfibre porosity is a vital factor in artificial scaffolds; high porosity can facilitate cell adhesion and increase cell proliferation. Hence, the cross-linked alginate scaffold may not be as useful as the alginate/MgO scaffolds for tissue engineering applications.

**Figure 3:**
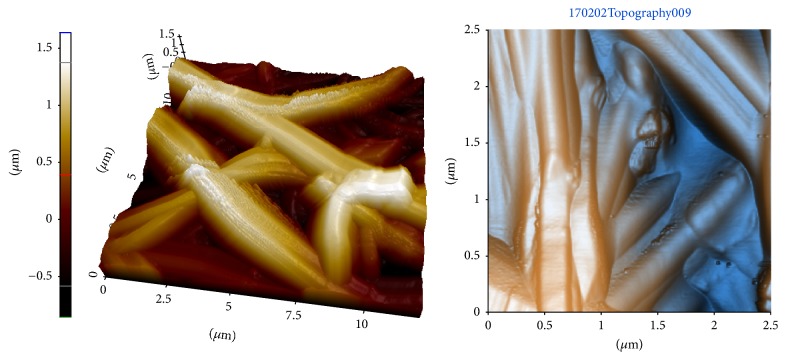
3D and 2D AFM images of alginate nanocomposite scaffolds.

In all cases, SEM images (Figures 2(a)–2(d)) and AFM images (Figure 3) reveal that the surfaces of the electrospun alginate-based fibres and alginate/MgO fibres are smooth (surface roughness (Ra) is 48 nm) and free of unusual artifacts that might suggest defects. On the other hand, some fillers, such as halloysite and ZnO, have been reported to affect the morphology of electrospun nanofibres—these fibres result in bead formation along the nanofibres [19, 35].

#### 3.2.2. FTIR Analysis

**Figure 4:**
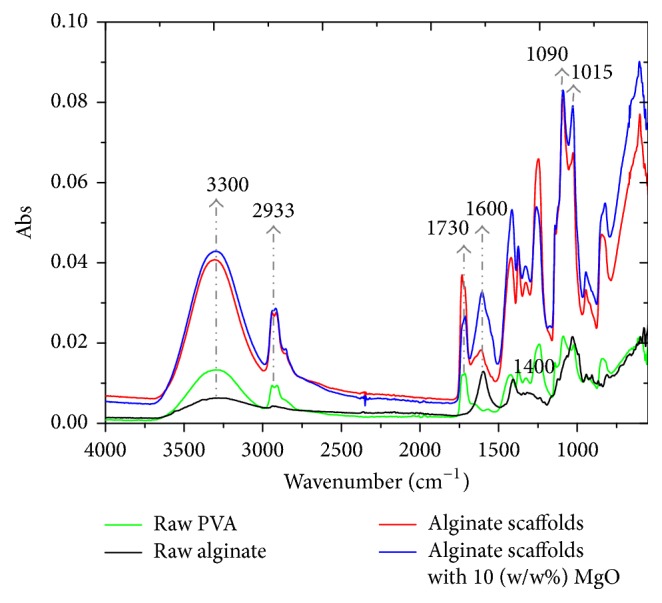
FTIR spectrum of raw alginate, raw PVA, and electrospun alginate-based scaffolds and alginate/MgO scaffolds.

Figure 4 shows the graphs of intensity versus wavenumber derived from FTIR analysis of alginate composite scaffolds. The results from raw alginate and MgO are also presented here for the purpose of comparison. The hydroxyl groups at 3300 cm^−1^, asymmetric carboxyl at 1600 cm^−1^, symmetric carboxyl at 1400 cm^−1^, and carbonyl functional groups at 1015 cm^−1^ [19] that appear in the FTIR spectra are attributed to the major functional groups of sodium alginate of the alginate scaffold (scaffolds contain alginate to PVA weight ratio of 3 : 2). Functional groups of PVA such as O-H stretching at 3300 cm^−1^, C-H stretching of alkyl groups at 2933 cm^−1^, C=O and C-O stretching of acetate groups at 1730 cm^−1^, and C-C stretching at 1090 cm^−1^ [34] can also be seen in the alginate-based scaffolds although many of these peaks overlap somewhat with the functional groups of alginate. The functional groups of MgO such as Mg-O stretching vibrational band at 540 cm^−1^ could not be isolated in the spectrum of nanocomposite scaffolds since those peaks overlap appreciably with the intense peaks of alginate and PVA. However, the presence of functional groups of alginate and PVA in the electrospun nanocomposite scaffolds confirms that the addition of MgO nanoparticles did not affect the structural integrity of the polymer blend.

#### 3.2.3. Tensile Properties

**Figure 5:**
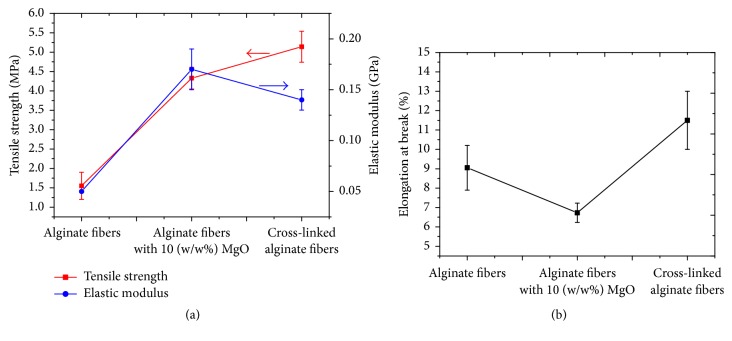
Plots of (a) tensile strength and elastic modulus and (b) elongation at break of electrospun alginate-based, alginate/MgO, and cross-linked alginate scaffolds.

Figure 5(a) shows the graph of tensile strength (*σ*_*U*_) and elastic modulus (*E*) of the respective alginate-based, alginate/MgO, and cross-linked alginate nanofibrous scaffolds. Statistical analysis (ANOVA) reveals that the means of *σ*_*U*_ of the respective scaffolds are significantly different (*P* < 0.05); the Tukey Post Hoc analysis reveals that this occurs between the alginate and alginate/MgO meshes. Similar conclusions have also been observed for *E*. Thus the addition of MgO nanoparticles results in enhanced mechanical properties. In particular, *σ*_*U*_ of the alginate/MgO mesh (mean *σ*_*U*_ = 4.33 MPa) is approximately three times greater than that of the alginate scaffold (mean *σ*_*U*_ = 1.55 MPa). Similarly, *E* of the alginate/MgO scaffold (mean *E* = 0.17 GPa) is three times greater than that of the alginate scaffold (mean *E* = 0.05 GPa). The increase in *σ*_*U*_ is attributed to the ability to transfer stress from the matrix phase to fillers, facilitated an efficient interaction at the filler-matrix interface, and is directed by hydrogen bonding between the abundant hydroxyl groups of alginate/PVA complex and MgO nanoparticles. Furthermore, these enhanced interfacial interactions between the fillers and matrix also contribute to high structural rigidity (i.e., *E*), as a result of restriction to the mobility of the polymer chains, when the scaffold deforms under an applied load. Of note, as expected *σ*_*U*_ of cross-linked alginate scaffold is higher than that of the untreated alginate scaffold. However, the cross-linked alginate scaffold is only marginally higher (i.e., 18%) than that of alginate/MgO scaffold. This could be due to the presence of covalent bonds associated with intramolecular and intermolecular interactions of PVA monomers [33]. Although cross-linked alginate scaffolds exhibit better tensile properties than MgO reinforced alginate nanocomposite scaffolds, cross-linking reduces the interfibre porosity of the scaffolds (Section 3.2.1). Consequently, this is unfavourable for promoting cell growth and proliferation as pointed out earlier. Therefore, nanofillers reinforced alginate/PVA scaffolds could be more advantageous than cross-linked alginate/PVA scaffolds for tissue engineering applications.

Figure 5(b) shows a plot of elongation at break (*ɛ*_*U*_) versus the respective alginate, alginate/MgO, and cross-linked alginate scaffolds. It is observed that *ɛ*_*U*_ of alginate/MgO scaffold is smaller than that of alginate scaffold (statistically significant, *P* < 0.05). This suggests that the addition of MgO nanoparticles to alginate-based matrix could contribute to a decrease in *ɛ*_*U*_ from 9.05% to 6.73%. This could be attributed to the increased rigidity of polymer chains as pointed out in previous paragraphs. However, *ɛ*_*U*_ of cross-linked alginate scaffold is slightly higher than that of the untreated alginate scaffold. This could be attributed to the formation of plasticizers from the left-over solvent in the scaffold (entrapped solvent in the fibres) after those were cross-linked with 0.5 M glutaraldehyde solution [21].

In order for an implanted scaffold to be able to provide the mechanical (as well as shape, see Section 3.2) stability to the tissue [36], the desired mechanical properties of the structure, that is, the processed scaffold, should be comparable to that of the host tissue. Thus, for the scaffold to be able to take up stress and not fail, that is, rupture, when an external load is acting on the tissue, the mechanical stability is regulated by the strength and extensibility of the scaffold. Table S2 (in supplementary data) lists the mechanical properties, namely, *E*, *σ*_*U*_, and *ε*_*U*_, of some soft connective tissues. With regard to strength, most tissues such as tendons, ligaments, and percardia possess fracture strengths that are at least one order of magnitude higher than what the alginate-based/MgO scaffolds could take, with the exception of the heart valve such as aorta and mitral leaflets. Thus, it would appear that the alginate-based/MgO scaffold is mechanically compatible, from the strength perspective, with the mitral heart valve leaflet as well as the aorta valve. However, it is noted that the extensitivity of these valves is about one order of magnitude higher than that of the alginate-based/MgO mesh. Nevertheless, as the meshes were tested in dry condition, it may be reasonable to anticipate that the extensibility of the alginate-based/MgO mesh could be appreciably higher when these are tested in wet condition. Of course, the alginate-based scaffolds (albeit a lower strength) may be a possible candidate for ECM-substitute scaffold as its extensibility could match those possessed by the valves.

#### 3.2.4. Thermal Properties

**Figure 6:**
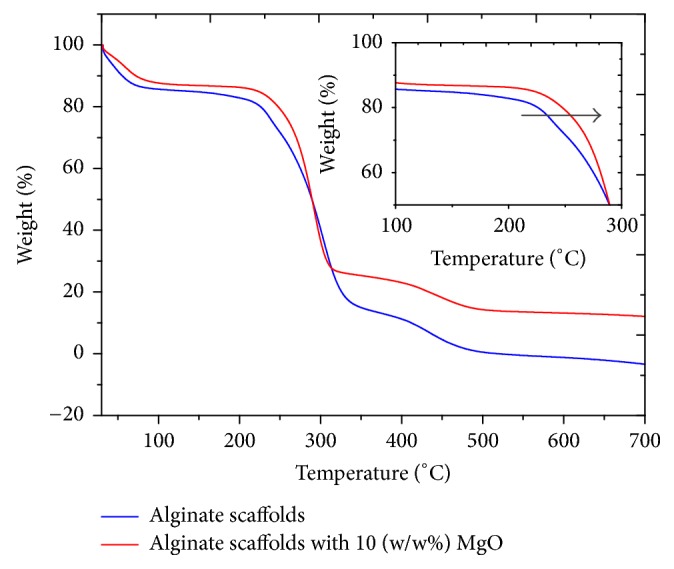
Thermogravimetric curves of electrospun alginate and alginate/MgO scaffolds.

Figure 6 shows plots of mass of the scaffold versus temperature derived from TGA for the respective alginate and alginate/MgO scaffolds. In all cases, initially the mass of the mesh decreases steadily with increasing temperature until at around 50°C. Between 50°C and 250°C, the mass shows no appreciable change with increasing temperature; beyond 250°C the mass decreases drastically. The drastic decrease in mass appears to follow a two-stage process: the first stage corresponds to a rapid decrease in mass while the second stage reveals a less rapid decrease in mass. Thereafter, at about 500°C, the mass of the alginate scaffold is almost zero, implying that the mesh is completely burnt off. On the other hand, the mass of the alginate/MgO scaffold is observed to be equal to 12%, suggesting that some residues including MgO are left behind.

The initial mass loss of about 10–12 wt% (at around 100°C) could be attributed to the removal of entrapped moisture and left-over solvent. The second mass loss at 200–300°C could be due to the thermal decomposition of polymer chains of alginate and PVA. Backbone structure of alginate decomposes at around 250°C due to the degradation of C-H bonds and C-O-C glycoside bonds in the main polysaccharide chain as a result of dehydration of saccharide chains [37]. More interestingly, the temperature of decomposition at 30% mass loss of the alginate/MgO occurs at around 272°C while that of alginate-based scaffold occurs at around 255°C. Thus, the thermal stability of the alginate/MgO scaffold is higher than that of alginate-based scaffold, that is, at 30% mass loss. These marginal improvements in thermal stability could be attributed to the high thermal stability of MgO nanoparticles which thermally decompose at around 2800°C.

## 4. Conclusion

An electrospinning method has been developed to fabricate alginate-based nanofibrous scaffolds, reinforced by MgO nanoparticles (10% (w/w)). The MgO nanoparticles were separately synthesised using a polymer template-based ex situ technique to achieve a near-spherical shape with an average diameter of 45 nm. For the purpose of comparison, the alginate scaffold, as well as glutaraldehyde cross-linked alginate scaffold, was fabricated. The mechanical properties of the alginate/MgO scaffold exhibit the highest tensile strength (*σ*_*U*_) and elastic modulus (*E*) among the three different types of scaffolds while retaining the interfibre porosity. The alginate/MgO scaffold yielded randomly oriented, ultrafine, virtually defect-free alginate nanofibres with a diameter ranging from 60 to 250 nm, similar to alginate scaffolds, and pore size is estimated to be 2–50 *μ*m. On the other hand, the cross-linked alginate scaffolds result in densely packed and extensively fused fibres; the resultant scaffolds also exhibit low interfibre porosity (compared with untreated alginate scaffolds). Altogether these suggest that the alginate-based/MgO scaffold is a suitable candidate for further investigation to be utilized as an artificial substitute for extracellular matrix in tissue engineering applications.

## Supplementary Material

Supplementary data comprises of macroscopic and microscopic images of electrospun alginate-based scaffolds to demonstrate their apparent properties as well as the morphological behaviour upon prolong cross-linking treatments. Furthermore, the section provides a numerical approach to determine the required filler amount to reinforce the scaffolds effectively. It also provides literature on the mechanical properties of tissues to compare the suitability of the fabricated scaffolds.

## Figures and Tables

**Table 1:** The optimised values of the operating parameters for spinnability and brief highlights of the morphology of the fibres in the scaffolds.

Fibre composition	Viscosity (P), flow rate (*μ*L/min), and voltage (kV)	Observed morphology
Alginate	68.4 p ± 0.08, 8–10, and 26	Diameter of 62–180 nm; randomly oriented and continuous; ultrafine, wavy, and smooth surface; beads-free
Alginate/MgO 10% (w/w)	72.2 p ± 0.0, 8–10, and 26	Diameter of 83–230 nm; randomly oriented and continuous; ultrafine, wavy, and smooth surface; beads-free
