# Supplementary material for: Magnesium Oxide Nanoparticles Reinforced Electrospun Alginate-Based Nanofibrous Scaffolds with Improved Physical Properties
Source: Int J Biomater. 2017 Jun 11;2017:1391298. doi: 10.1155/2017/1391298 (PMC5485316; doi:10.1155/2017/1391298)
Supplement: Supplementary file 1 — Supplementary data comprises of macroscopic and microscopic images of electrospun alginate-based scaffolds to demonstrate their apparent properties as well as the morphological behaviour upon prolong cross-linking treatments. Furthermore, the section provides a numerical approach to determine the required filler amount to reinforce the scaffolds effectively. It also provides literature on the mechanical properties of tissues to compare the suitability of the fabricated scaffolds. [file 1391298.f1.docx]

**Supplementary Data**

**S1. Morphology of Electrospun Alginate Scaffolds**


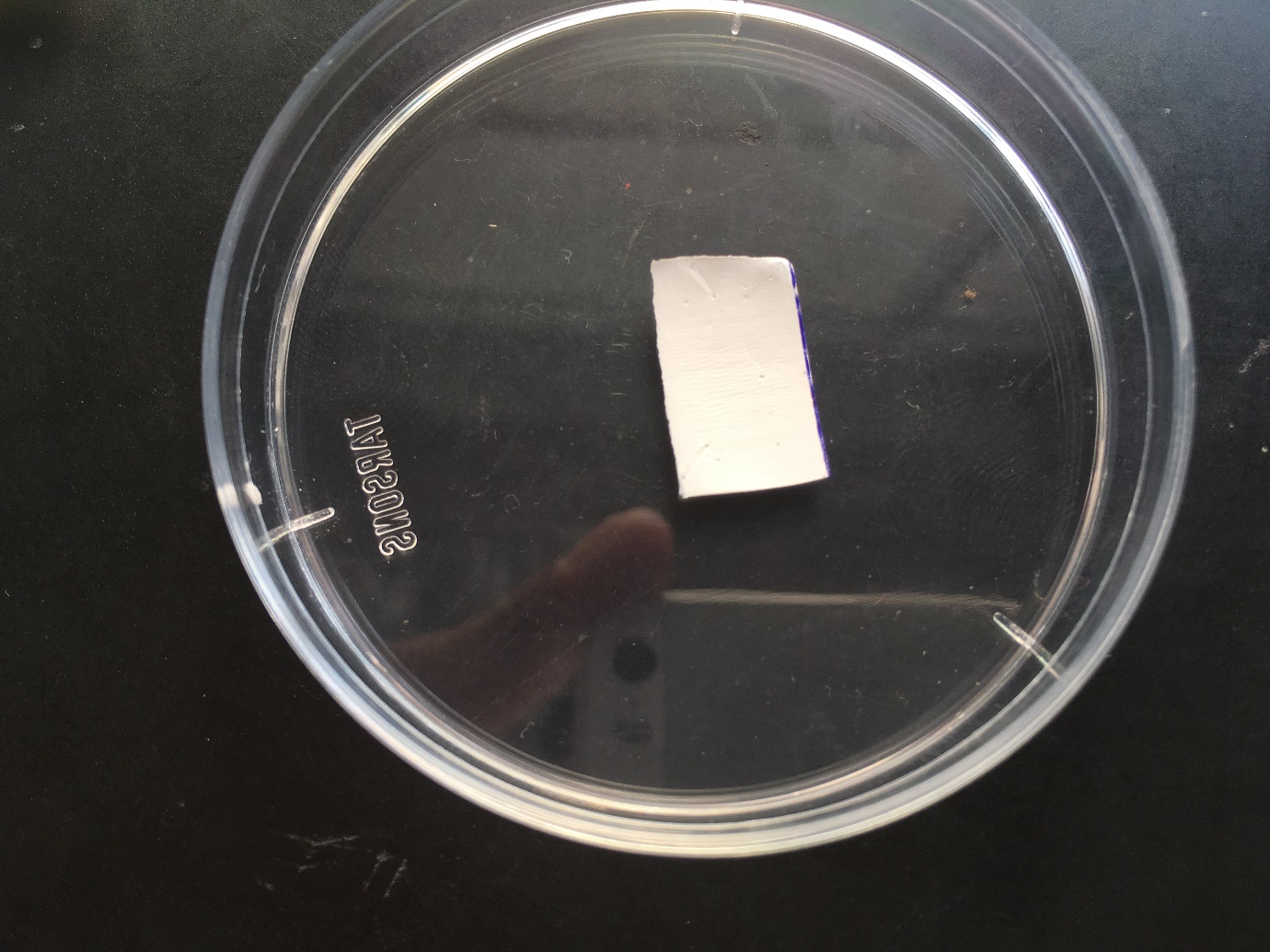


**2 cm**


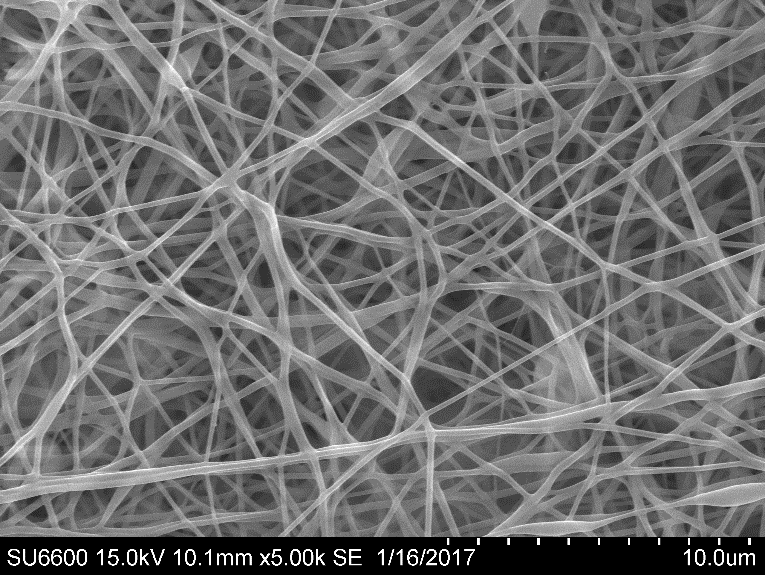


Fig. S1: Apparent look of electrospun alginate nanofibrous scaffolds


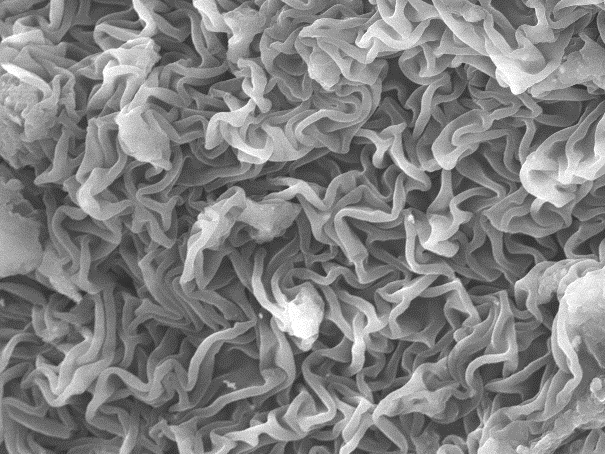

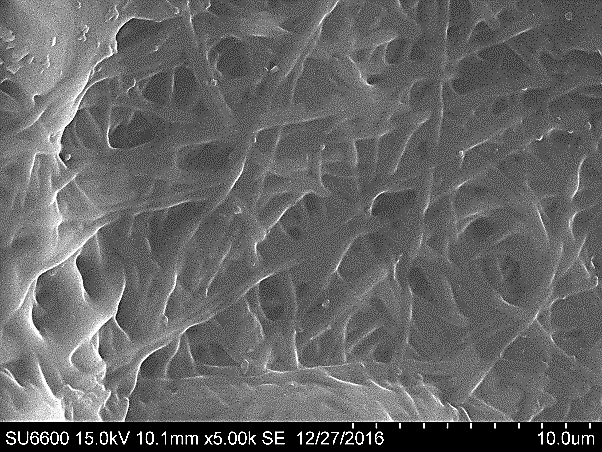


Fig. S2: SEM images of 2 hours cross-linked and 24 hours cross-linked electrospun alginate scaffolds

**S2. Scaffold Design**

This section aims to numerically determine the required MgO loading which provides desired mechanical properties. With regard to the MgO particles reinforcing the alginate-based scaffolds, one key consideration concerns the concentration of MgO particles, which may be parameterised by the volme fraction of the particles (*V*_MgO_) (i.e. the alternative of the % w/w). In most cases, the mechanical properties of these particle reinforced composites may be tuned by varying the concentration of the nanoparticles, but there exist a critical level of the volume fraction beyond which the diminution of the mechanical properties predominates with increasing volume fraction [1-3]. For this initial study, the intent is to provide simple order-of-magnitude estimates of the critical volume fraction, *V*_min_—corresponding to the highest value for the *E* and *σ*_U_—resulting from several different physical processes, namely elastic stress transfer and plastic stress transfer.

To begin, according to elastic stress transfer process, *E*′_MgO_ (= *E*_MgO_[1-tanh(β)/β]) is the modified stiffness of the MgO particles in the composite, where β=*q*√([*G*_m_/*E*_MgO_]{2/ln(*P*_MgO_/*V*_MgO_)}), *q* the particle aspect ratio (≈1), and *P*_MgO_ the particle packing factor [4]. Let *E*′_m_ (=dσ_m_/dε_m_) be the stiffness of the alginate/PVA matrix material when it is in a state of plasticity, where σ_m_ and ε_m_ are the respective average stress and strain in the matrix, at a predetermined composite strain [4]. It follows that the *E* can be estimated to order of magnitude using the modified rule-of-mixture for elastic modulus of the scaffolds, i.e. *E* = *E*′_MgO_*V*_MgO_+*E*′_m_[1-*V*_MgO_], for *V*_MgO_ ≤ *V*_min_ [4]. When this condition is satisfied, this results in the simple linear dependence of *E* on *V*_MgO_, underlying the effectiveness of the MgO particles for taking up stress in the composite. On the other hand, if *V*_MgO_ > *V*_min_, aggregates of particles, held by weak forces (e.g. van Der Waals forces) could form in the composite during the processing stage, and could increase in number and size, with increase in *V*_MgO_ [4]. The effective stiffness of the particle aggregates could be much smaller than the *E*_MgO_. Consequently, an estimate of *E* could go by *E* ≈ *E*_m_[1-*V*_MgO_] (i.e. when *V*_MgO_ > *V*_min_). On this simple argument, it follows that *V*_MgO_ (=*V*_min_) can be estimated to order of magnitude by evaluating *E* = *E*′_MgO_*V*_MgO_+*E*′_m_[1-*V*_MgO_] ≈ *E*_m_[1-*V*_MgO_], giving

| *V*_min_ = [*E*_m_ - *E*′_m_]/{*E’*_MgO_ + [*E*_m_ - *E*′_m_]}. | (1) |
| --- | --- |

To determine *V*_min_, although one may assume that *E*′_m_ < *E*_m_, numerically *E*′_m_ could be comparable to 99.5% of *E*_m_ (= 100 Pa; [5]). For simplicity, *P*_MgO_ is identified with the triangular edge packing factor (=2π/√3) [6], *G*_m_ may be set to 10 Pa [5], *E*_MgO_ may be set to 100 GPa [7], and the β expression is evaluated for *V*_MgO_ ranging from 0.005 to 0.100; this enables us to plot a graph of *V*_min_ versus *V*_MgO_ (Fig. 1), where a possible solution can be identified with *V*_min_ = *V*_MgO_. We find that this occurs at *V*_min_ ≈ 0.03. Experimentally, the *V*_min_ ≈ 0.03 would then correspond to a 10 % w/w of MgO. Table S1 summarizes the values of the respective mechanical and structural parameters used in this calculation.

The *V*_min_ for the σ_U_ is based on a slightly different argument. Hence, the *V*_min_ for the σ_U_ may be different from the *V*_min_ of the *E* [3]. It follows that the σ_U_ can be estimated to order of magnitude using a modified rule-of-mixture for the fracture strength of the scaffolds, i.e. σ_U_ = σ_MgO,U_*V*_MgO_+σ_m_[1-*V*_MgO_], where σ_m_ is the average stress in the alginate/PVA matrix at the fracture strain of the particle. This is only applicable when the *V*_MgO_ is smaller than the *V*_min_. For *V*_MgO_ > *V*_min_, the σ_U_ is approximated by σ_U_ ≈ σ_m,U_{1-*V*_MgO_}. Thus, the *V*_min_ may be found by equating the σ_U_ from the two expressions, i.e. σ_U_ = σ_MgO,U_*V*_MgO_+σ_m_[1-*V*_MgO_] ≈ σ_m,U_{1-*V*_MgO_}, giving

| *V*_min_ = [σ_m,U_ - σ_m_]/{ σ_MgO,U_ + [σ_m,U_ - σ_m_]}. | (2) |
| --- | --- |

According to Uchida et al., σ_MgO,U_ ≈ 700 MPa [8]; according to Tonsomboon et al., σ_m,U_ ≈ 50 MPa [9]. To order of magnitude, σ_m_ goes by one-half of σ_m,U_. It follows that the prediction for the largest fracture strength requires *V*_min_ = 0.03; this turns out to be comparable with the prediction of *V*_min_ for the *E*. Table S1 summarizes the values of the respective mechanical and structural parameters used in this calculation.


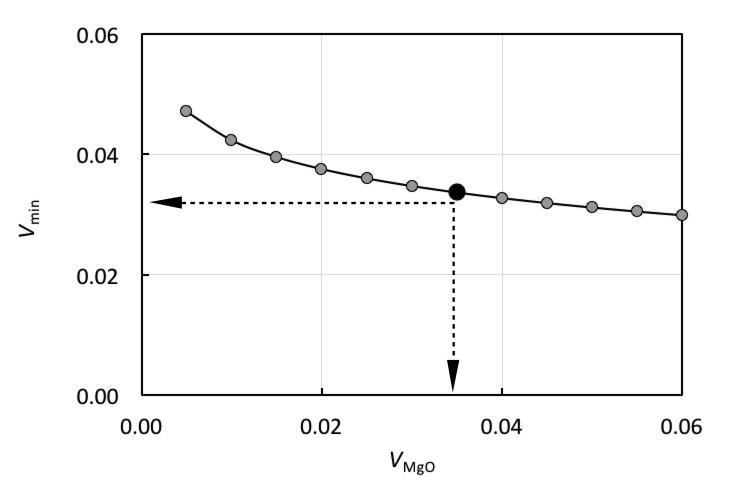


Fig. S3: Graph of V_min_ versus V_MgO_. The point on the curve where V_min_ ≈ V_MgO_ is indicated by a circle with a darker shade.

Table S1. Parameters used in the prediction of an optimised alginate-based/MgO scaffolds

| Parameters | Value | Reference |
| --- | --- | --- |
| *P*_MgO_ | 2π/√3 | [6] |
| *E*_m_ | 100 MPa | [5] |
| *G*_m_ | 10 MPa | [5] |
| *E*_MgO_ | 100 GPa | [7] |
| σ_MgO,U_ | 700 MPa | [8] |
| σ_m,U_ | 50 MPa | [9] |

**S3. Comparison of mechanical properties**

Table S2: Mechanical properties of some tissues

| **Tissue type** | ***E* (MPa)** | **σ_U_ (MPa)** | **ε_U_** | **Literature** |
| --- | --- | --- | --- | --- |
| Aorta valve, human | 2-15 | 0.4-2.6 | 0.22-0.30 | [10, 11] |
| Mitral valve anterior leaflet | 3.6±1.8 | - | - | [12] |
| Mitral valve anterior leaflet | - | 0.05-0.45^Anterior^; 0.10-0.80^Posterior^ | - | [13] |
| Mitral valve chordae tendinese, human* | 329.6±227.8 to 387.7±289.6 | 36.8±22.5 to 40.8±24.6 | 0.20±0.09 to 0.21±0.12 | [14] |
| Tendon fascicle, mouse tail ^∀^ | 417.1±51.3 to 688.6±28.8 | 26.1±3.0 to 61.8±3.3 | - | [15] |
| Anterior cruciate ligament fascicle, sheep^⊗^ | 300 | 40 | 0.24 | [15, 16] |

^#^ : from small strain (<0.2) to large strain (>0.2); * grouped by leaflet type; ^∀^ 1.6 months old to 29.0 months old; ^Δ^ from axial to transverse direction with respect to the spine; ^⊗^ average value

**Reference**

[1] De Silva RT, Pasbakhsh P, Goh KL, Chai S-P, Ismail H. Physico-chemical characterisation of chitosan/halloysite composite membranes. Polym Test. 2013;32(2):265-271.

[2] De Silva RT, Pasbakhsh P, Goh KL, Mishnaevsky Jr L. 3-D computational model of poly (lactic acid)/halloysite nanocomposites: Predicting elastic properties and stress analysis. Polym. 2014;55(24):6418-6425.

[3] Chew SL, Wang K, Chai SP, Goh KL. Elasticity, thermal stability and bioactivity of polyhedral oligomeric silsesquioxanes reinforced chitosan-based microfibres. J Mater Sci Mater Med. 2011;22(6):1365.

[4] Goh KL. Discontinuous-Fibre Reinforced Composites: Fundamentals of Stress Transfer and Fracture Mechanics: Springer; 2016.

[5] Banerjee A, Arha M, Choudhary S, Ashton RS, Bhatia SR, Schaffer DV, et al. The influence of hydrogel modulus on the proliferation and differentiation of encapsulated neural stem cells. Biomaterials. 2009;30(27):4695-4699.

[6] Mohonee VK, Goh KL. Effects of fibre–fibre interaction on stress uptake in discontinuous fibre reinforced composites. Compos Part B Eng. 2016;86:221-228.

[7] Zha C-S, Mao H-k, Hemley RJ. Elasticity of MgO and a primary pressure scale to 55 GPa. Proceedings of the National Academy of Sciences. 2000;97(25):13494-13499.

[8] Uchida T, Wang Y, Rivers ML, Sutton SR. Yield strength and strain hardening of MgO up to 8 GPa measured in the deformation-DIA with monochromatic X-ray diffraction. ‎Earth Planet Sci Lett. 2004;226(1–2):117-126.

[9] Tonsomboon K, Butcher AL, Oyen ML. Strong and tough nanofibrous hydrogel composites based on biomimetic principles. Mater Sci Eng: C. 2017;72:220-227.

[10] Hasan A, Ragaert K, Swieszkowski W, Selimović Š, Paul A, Camci-Unal G, et al. Biomechanical properties of native and tissue engineered heart valve constructs. J Biomech. 2014;47(9):1949-1963.

[11] Balguid A, Rubbens MP, Mol A, Bank RA, Bogers AJ, Van Kats JP, et al. The role of collagen cross-links in biomechanical behavior of human aortic heart valve leaflets—relevance for tissue engineering. Tissue Eng. 2007;13(7):1501-1511.

[12] Liao J, Yang L, Grashow J, Sacks MS. The relation between collagen fibril kinematics and mechanical properties in the mitral valve anterior leaflet. J Biomech Eng. 2007;129(1):78-87.

[13] Weinberg EJ, Kaazempur-Mofrad MR. A large-strain finite element formulation for biological tissues with application to mitral valve leaflet tissue mechanics. J Biomechanics. 2006;39(8):1557-1561.

[14] Zuo K, Pham T, Li K, Martin C, He Z, Sun W. Characterization of biomechanical properties of aged human and ovine mitral valve chordae tendineae. J Mech Behav Biomed Mater. 2016;62:607-618.

[15] Goh K, Holmes D, Lu H-Y, Richardson S, Kadler K, Purslow P, et al. Ageing changes in the tensile properties of tendons: influence of collagen fibril volume fraction. J Biomech Eng. 2008;130;021011.

[16] Yeo YL, Goh KL, Kin L, Wang HJ, Listrat A, Bechet D. Structure-property relationship of burn collagen reinforcing musculo-skeletal tissues. Key Eng Mater. 2011;478;87-92.
